# Synthesis and Characterization of New Ferrite-Lignin Hybrids

**DOI:** 10.3390/polym13152495

**Published:** 2021-07-28

**Authors:** Iuliana Spiridon, Ioan-Andrei Dascalu, Adina Coroaba, Irina Apostol, Mircea Nicolae Palamaru, Alexandra Raluca Iordan, Adrian Iulian Borhan

**Affiliations:** 1 “Petru Poni” Institute of Macromolecular Chemistry, Grigore Ghica–Voda 41, 700487 Iasi, Romania; idascalu@icmpp.ro (I.-A.D.); adina.coroaba@icmpp.ro (A.C.); apostol.irina@icmpp.ro (I.A.); 2 Faculty of Chemistry, Alexandru Ioan Cuza University of Iaşi, 11, Carol I Blvd., 700506 Iasi, Romania; palamaru@uaic.ro (M.N.P.); alexandra.iordan@uaic.ro (A.R.I.); adrian.borhan@uaic.ro (A.I.B.)

**Keywords:** ferrite, lignin, hybrids, synthesis, characterization

## Abstract

The paper presents the synthesis and characterization of new cobalt ferrite-lignin hybrids. The hybrids were obtained through the combustion of cobalt nitrate and ferric nitrate, two kinds of lignin being used as combustion agents. The temperatures of calcination were 500 °C and 900 °C, respectively. The hybrids were characterized by Fourier transform infrared spectroscopy (FT-IR), X-ray powder diffraction (XRD), scanning electron microscopy (SEM), energy-dispersive X-ray (EDX), and X-ray photoelectron spectroscopy (XPS). The magnetic properties were also assessed by vibrating sample magnetometer system (VSM). This facile synthesis method made it possible to obtain cobalt ferrite-lignin hybrids with a spinel structure. Their particle sizes and crystallite sizes have increased with an increment in the calcination temperature. A different occupancy of cations at octahedral and tetrahedral sites also occurred upon the increase in temperature. The hybrids comprising organic lignin presented the best magnetic properties.

## 1. Introduction

Lignin represents the most abundant but underutilized natural polymer with aromatic structure, constituting up to 35% of wood biomass. It has a 3D network aromatic structure, which has not yet been fully defined. Lignin is the result of the biosynthesis process of three phenylpropanoid units named *p*-hydroxyphenyl (H), guaiacyl (G), and syringyl (S), derived from *p*-coumaryl, coniferyl, and sinapyl alcoholic precursors, respectively [1,2,3]. The proportion of these monomers varies as a function of the biomass source. Thus, hardwood lignin is composed of G and S units, as well as low levels of H units, while softwood lignin consists of G units and a small amount of H units. These building units are linked by different carbon–carbon and/or carbon–oxygen bonds, β-O-4′ and 5–5′ being the most abundant ones. It is worth mentioning that the elucidation of the lignin structure cannot be performed by the decomposition methods. The content of the functional groups (phenolic hydroxyl, alcoholic hydroxyl, and carboxyl groups) also depends on the pulping process applied for lignin separation. Biomass lignin mainly consists of carbon, hydrogen, and oxygen. After the delignification process, other chemical elements could also be present. Thus, lignins separated by kraft pulping and sulfite pulping could contain sulfur, while the presence of phosphor was reported in organosolv lignin, due to the delignification of wood with organic solvents [4].

In the last years, the interest in the lignin generated as a by-product of the pulping process has increased greatly, due to the societal and environmental needs to replace oil-based polymeric materials. In spite of its poor processability, brittleness, structural heterogeneity and low reactivity, lignin is already being used as controlled release system, adsorbent, dispersant, bioplastic, composites component, and a source of carbon fibers [5,6,7,8,9,10,11,12,13], and represents a material with a huge potential for multiple industrial applications. From the green chemistry perspective, transforming lignin into valuable products with various applications represents a great challenge. The production of value-added chemicals from biomass is one of the most important value chains within the emerging bio-economy. At present, lignin is mainly used for energy production by combustion, but it can be a precursor of activated carbon with a hierarchical porous structure. In this work, we are interested in turning it into a component of hybrid materials. The valorization of lignin through the development of hybrid bio-inorganic materials, with dual functionalities, is a sustainable method of biomass waste management.

Cobalt ferrite (CoFe_2_O_4_) is one of the most important spinel materials, which is widely studied due to its adequate properties (high coercivity, wear resistance, electrical insulation, moderate saturation magnetization, and stability) for interactions with the environment [14,15]. Spinels are synthesized through different chemical methods, such as solid-state reactions, mechanochemical synthesis, co-precipitation, microemulsion synthesis, hydrothermal synthesis, or solution combustion methods. In the last method, different organic compounds are used to obtain ferrite spinel structures [16], which are promising for different technological [17] and biomedical applications [18,19].

Numerous spinel ferrite composites have been developed [20,21,22] to be used in the treatment of polluted waters. Recently, pine sawdust and waste batteries have been used to obtain a low-cost material [23] exhibiting a stable adsorption activity after the sixth use. Although the spinel cobalt ferrites have been studied by several groups [24,25], the uniform dispersion of magnetic particles into a polymeric matrix is quite difficult to achieve via the conventional mixing of magnetic particles and polymers, because of both magnetic moments and van der Waals forces that result in the agglomeration of particles, and there is much more to explore about applying spinel ferrites in a polymeric matrix.

To the best of our knowledge, no information on the cobalt ferrites-lignin hybrids’ synthesis and the characterization of cobalt ferrites-lignin hybrids has been reported so far. In this article, for the first-time, we report the direct synthesis of cobalt ferrite-lignin hybrids, starting from organosolv and kraft lignin and the nitrates of Co and Fe.

The obtained materials were characterized by Fourier transform infrared spectroscopy (FT-IR), X-ray diffraction (XRD), X-ray photoelectron spectroscopy (XPS), and SEM (scanning electron microscopy) techniques. The dynamic vapor sorption capacity and the magnetic properties of the developed hybrids were also evaluated.

## 2. Materials and Methods

### 2.1. Materials

Cobalt nitrate (Co(NO_3_)_2_·6H_2_O) and ferric nitrate (Fe(NO_3_)_3_·9H_2_O), of analytical purity grade, were purchased from Sigma-Aldrich (Darmstadt, Germany) and used as cation sources. 

Organosolv lignin (LO) extracted with acetic acid/phosphinic acid from birch wood [4], as well as Lignoboost^®^ (from from Innventia’s pilot plant in Bäckhammar in Sweden) lignin (LB) from kraft cooking of softwood [26], were used as as the chelating/combustion agents. 

### 2.2. Synthesis

**Scheme 1:**
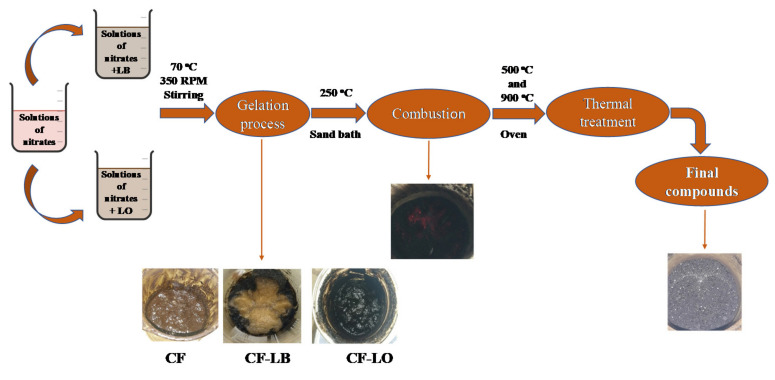
Schematic illustration of the ferrite-lignin hybrids’ obtainment.

The atomic ratio of the metal cation Co^2+^: Fe^3+^ was 1:2 and the mass ratio for ferrite: lignin was 1:3. All the components of the reaction system were dissolved in distilled water. The precursor solutions were placed on a magnetic stirrer after mixing. The temperature was set at 70 °C. During magnetic stirring at 350 rpm, a brown gel was obtained. The dried gels were ignited on a hot plate by increasing the temperature up to 250 °C. After the flame was extinguished, the samples were thermally treated at 500 °C and 900 °C, respectively (Scheme 1). 

The samples were named according to the chelating/combustion agents used and the treatment temperatures, as follows: CF-LB500, CF-LB900, CF-LO500, and CF-LO900. 

### 2.3. Characterization

#### 2.3.1. FTIR Spectra

The infrared absorption spectra (FTIR) of the materials were recorded within the range of 400–4000 cm^−1^ with a resolution of 2 cm^−1^ and 32 scans, at room temperature, using a Bruker Vertex 70 FTIR spectrometer (Bruker, Marne-la-Vallée, France).

#### 2.3.2. X-ray Diffraction

X-ray diffraction analysis was performed on a Rigaku Miniflex 600 diffractometer (Tokyo, Japan) using CuKα-emission in the angular range of 10–80° (2θ) with a scanning step of 0.01° and a recording rate of 1°/min. The phase identification was accomplished using the Crystallography Open Database (COD) data and the SmartLab II software package for powder X-ray diffraction analysis. Each sample diffractogram was subjected to a whole powder pattern fitting calculation (using the Rietveld method) with the CIF structure file COD ID no. 1533163. 

#### 2.3.3. Particle Size Distribution

The particle size distribution was evaluated using the dynamic light scattering analysis (Zetasizer model Nano ZS, with red laser 633 nm He/Ne; Malvern Instruments, Malvern, UK). The determinations were made on a 2 mL sample of 0.5% ferrite lignin solution without dilution. All measurements were carried out at 25 °C.

#### 2.3.4. Dynamic Vapour Sorption (DVS) Capacity

An IGAsorp apparatus (Hiden Analytical, Warrington, UK) was used to measure sorption capacity in the dynamic regime. At each 10% humidity step, the vapor pressure increased with a pre-established equilibrium time of 10 min. Before sorption, the samples were dried in flowing nitrogen (250 mL min^−1^) until the weight was constant at a RH < 1%. Based on isothermal studies, the specific surface area was calculated by means of the Brunauer-Emmett-Teller Equation (1):

(1) $$W=\frac{W_{m}\times C\times \frac{p}{p_{0}}}{\left(1-\frac{p}{p_{0}}\right)\times \left(1-\frac{p}{p_{0}}+C\times \frac{p}{p_{0}}\right)}$$

where *p* and *p*_0_ are the equilibrium and the saturation pressure of adsorbates at the temperature of adsorption, *W* is the quantity of water adsorbed, and *W_m_* is the monolayer quantity of water adsorbed, while *C* is the BET constant. The BET model was used for calculating the surface area of the materials by the physical adsorption of water vapours. The data are presented as an average of the three tests 

#### 2.3.5. Scanning Electron Microscopy

The surface morphology of the materials was investigated by (SEM). A (ESCM) Quanta 200 (Brno, Czech Republic) device (operated at an accelerating voltage of 20 keV), equipped with an Energy Dispersive X-ray (EDX) module, was used. 

#### 2.3.6. X-ray Photoelectron Spectroscopy

(*XPS)* was performed on an Axis Nova device (Kratos Analytical, Manchester, UK), using AlKα radiation, with a 20 mA current and 15 kV voltage (300 W), and a base pressure of 10^−8^ to 10^−9^ Torr in the sample chamber. The XPS survey spectra for the samples were collected in the range of −5 ÷ 1200 eV, with a resolution of 1 eV and a pass energy of 160 eV. The high resolution spectra for all the elements identified from the survey spectra were collected using a pass energy of 20 eV and a step size of 0.1 eV. The data were analyzed using the ESCApe software. The binding energy of the C 1s peak was normalized to 285 eV.

#### 2.3.7. Magnetic Measurements

Magnetic measurements were performed using an AGM & VSM Magnetometer, Princeton Measurement Co. (Princeton, USA), under standard conditions. The maximum values of the magnetization (*M*_max_) and remanent magnetizations (*M*_r_) were evaluated.

## 3. Results

### 3.1. FTIR Spectra

**Figure 1:**
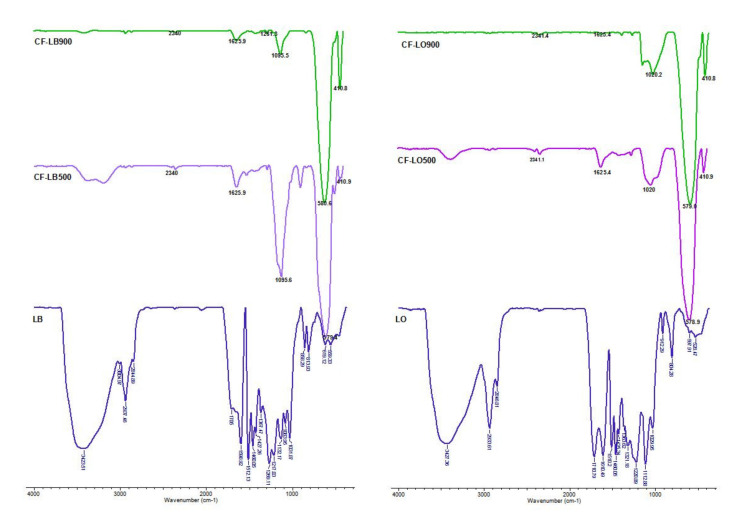
FTIR spectra of lignin hybrids.

For all cobalt ferrite-lignin hybrids, the presence of low-intensity peaks in the fingerprinting region of 900 to 650 cm^−1^ [27] suggests the occurrence of the poly-substituted phenolic structure of lignin (Figure 1). The absorption bands present at about 685 cm^−1^ and 410 cm^−1^ were due to the stretching vibrations of metal oxide in the octahedral group complex Co(II)–O^2−^ and Fe(III)–O^2−^ tetrahedral group complex of the cobalt ferrite phase, respectively, which proves the existence of spinel ferrite [28]. A typical low frequency band at around 585 cm^−1^, caused by the motion of oxygen with respect to the cations in the octahedral (O_h_) sites and characteristic of the spinel structure, was recorded [29]. 

The characteristic peaks of lignin are found in the ferrite-lignin spectra. Thus, the peaks at 2935 cm^−1^ and 2842.9 cm ^−1^, ascribed to the C-H stretching vibrations in lignin, are shifted to 2922.04 and 2852.6 cm^−1^, respectively, and are observed in all samples comprising lignin and cobalt ferrite. The peak intensities decreased as the calcination temperature increased to 900 °C, suggesting that the hydrogen molecules were removed. The peaks at 1625.9 and 1631.7 cm^−1^, corresponding to C=O stretching (conjugated), are present in the spectra of the hybrid materials. An important peak at 1382 cm^−1^, which can be attributed to aliphatic C–H in the methyl groups, was also recorded for the hybrid materials, as well as the peak at 1261 cm^−1^ specific to G ring breathing with carbonyl stretching. The peaks at 1095 cm^−1^ and 1020 cm^−1^, specific to the aromatic C-H deformation of S units and the C-O deformations of secondary alcohols and aliphatic ethers aromatic C-H in-plane deformation, respectively, are presented in the spectra of hybrids [30,31]. The band at 1261.4 cm^−1^ could be attributed to the aromatic C–O stretching of S- units and/or the condensed G- units at G_5_ in lignin, and it is present in all of the hybrids. The peaks recorded for all the hybrids at wavenumbers from 2340 to 2360 cm^−1^ are related to OH stretching from strong H-bonded-COOH [32].

Based on the findings discussed above, it can be concluded that the process of the synthesis of ferrite-lignin hybrids was realized. 

### 3.2. XRD

**Figure 2:**
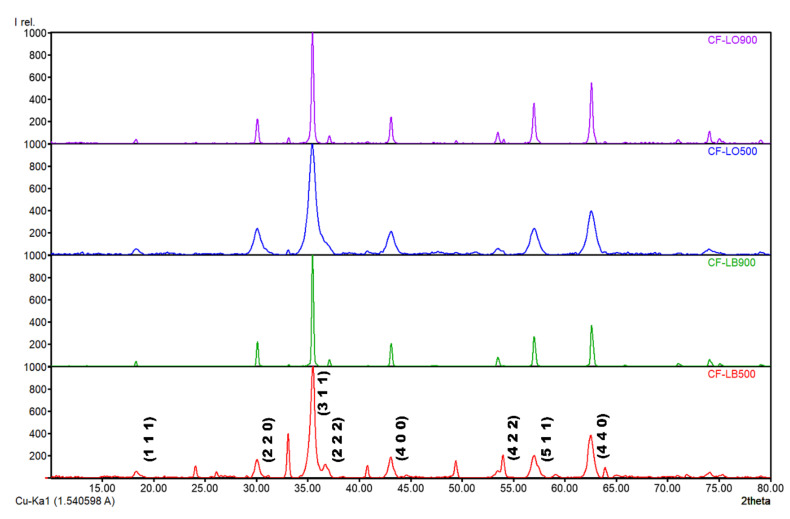
XRD spectra of lignin hybrids.

The crystalline structure of synthesized and thermally treated CoFe_2_O_4_-lignin hybrid particles was evaluated by XRD. The X-ray diffraction patterns of the materials are shown in Figure 2. All of the diffraction peaks are well-matched with the assigned phase-ferrite cobalt (COD entry no. 1533163). The seven two-theta values (30.08, 35.43, 37.05, 43.05, 53.44, 56.9, and 62.58) corresponding to the reflections of (220), (311), (222), (400), (422), (511), and (440) planes, confirm the formation of cobalt ferrite-based materials with a spinel structure. 

The corresponding patterns of the cobalt ferrite-lignin hybrids obtained at 900 °C present sharper and narrower peaks, revealing an improved crystallinity as a result of the atomic ordering promoted by the elevated calcination temperature. 

The phase assignment for each sample was verified using the whole-powder-pattern fitting calculation (Rietveld method) available with the instrument software. 

The parameters indicative of the refinement effectiveness Rwp, S, and *χ*^2^, were obtained for all the samples, and are in the range of 1.92–4.96% (Rwp), 0.8064–2.044 (S), and 0.6503–4.178 (*χ*^2^), respectively, showing a good fit between the measured and the calculated data (Table 1).

The data from Table 1 is evidence that the crystallite size, calculated based on Hall methods, increased with the calcination temperature. According to the literature data [33], a higher temperature promotes crystallization due to the atomic mobility, as well as the decrease in crystalline defects and internal strains. As result, crystal growth and the re-distribution of cations among octahedral and tetrahedral sites in the spinel structure are registered (see XPS data). The lattice parameter ‘*a*’ for lignin-hybrids is quite close to that of ferrite cobalt (COD entry no. 1533163), which is 8.3806 Å. The slow increase in the lattice constant with the increase in calcination temperature could be related to the variation in microstructure during thermal treatment, as well as to the ordering or reordering of cations in the cubic spinel structure [34], which has been confirmed by XPS data.

### 3.3. Particles Size Distribution

**Figure 3:**
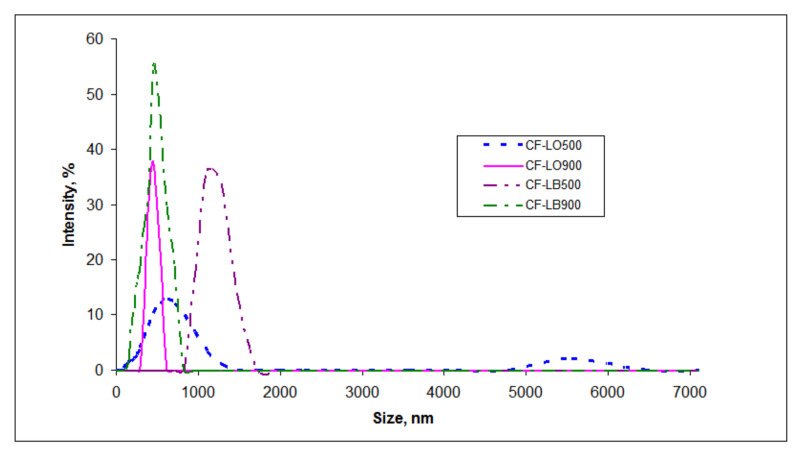
Particle size distribution of ferrite-lignin hybrids.

All of the hybrids suspensions were relatively monodisperse; the analysis indicated the presence of a unimodal size distribution (Figure 3) with polydispersity indices of 1.00 for CF-LO900 and 0.539 for CF-LO500. The hybrids comprising organic lignin recorded closer values, as follows: 0.968 for CF-LB900 and 0.598 for CF-LB500. The observed increase in the polydispersity indices with the increase in calcination temperature reveals good stabilization of the ferrite-lignin particles.

### 3.4. DVS

The data from Table 2 are evidence that the rise of calcination temperature from 500 to 900 °C has influenced the water sorption capacity of all the lignin hybrids, this parameter decreasing by approximately 5%. It was found that the hybrids obtained at 500 °C presented the lowest pore sizes, which is indicative of a high surface area of the particles. The increase in the average particles, size, following the rise in the calcination temperature, is clearly visible. The average pore size increased at 900 °C, probably due to the faster oxidation of organic components, which disappeared, and the shrinking volume. The large particles may be associated with particle growth from the smaller particles, or with the agglomeration of small particles [35]. Numerous voids between the particles are formed, which makes the particles loose and contributes to the formation of pores [36]. The specific surface area of the hybrids, calculated using the Brunauer-Emmett-Teller (BET) method, significantly decreased when increasing the calcination temperature. 

It could be due to the reinforcement of the hybrid structure as a result of the combination of the lignin fragments with ferrite, and the blocking of some of the its active sites [37]. Other authors reported a similar behavior upon the formation and crystallization of cobalt ferrite into a SiO_2_ sol-gel matrix [38].

### 3.5. SEM

**Figure 4:**
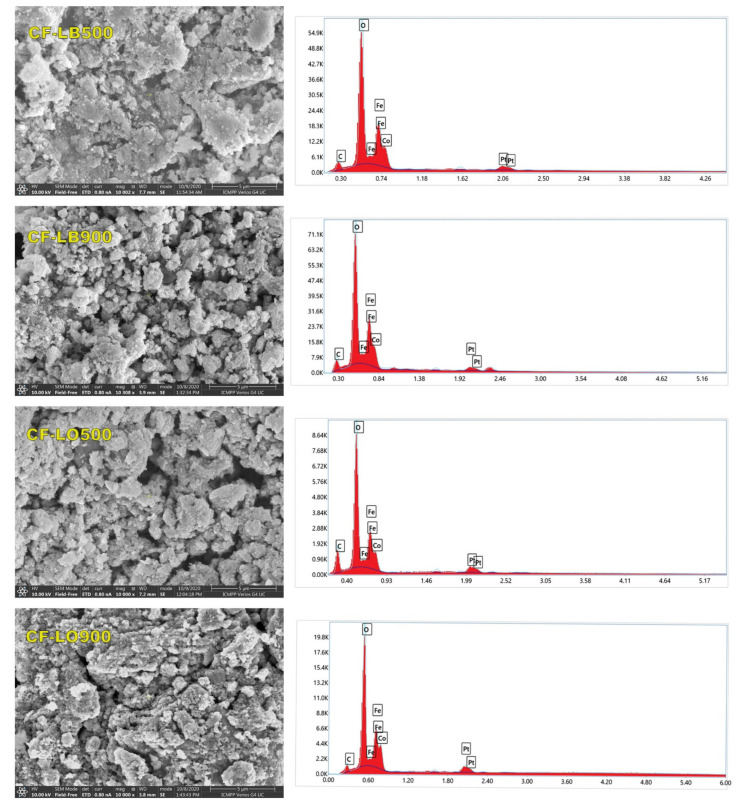
SEM micrographs (×10,000 magnification) and EDX spectra of hybrids samples.

The morphological aspects and the elemental distribution mapping of the materials were examined using a scanning electron microscope (SEM) equipped with an energy dispersive spectrometer (EDS). The micrographs (Figure 4) evidence that the material particles exhibit a polydisperse mesoporous aggregate. The voids and pores present in the hybrid samples could be attributed to the release of a large amount of gases during calcination.

The increase in the calcination temperature has resulted in the formation of various clusters and particle agglomerations. It is consistent with the results of XRD analysis. These agglomerations are due to the presence of magnetic interactions among the particles, as well as to the melting of small size nanoparticles which aggregated to form bigger particles in order to reduce the interfacial energy of the individual particles [39].

The energy dispersive spectroscopy of the samples evidenced O, C, Fe, and Co as the only present elements (Pt element coming from the sample preparation). Considering the weight percentage of the elements in the sample, it seems that the highest weight percentage is related to the oxygen element, which exists both in the cobalt ferrite and in the organic part (lignin) of the prepared hybrids.

### 3.6. XPS

**Figure 5:**
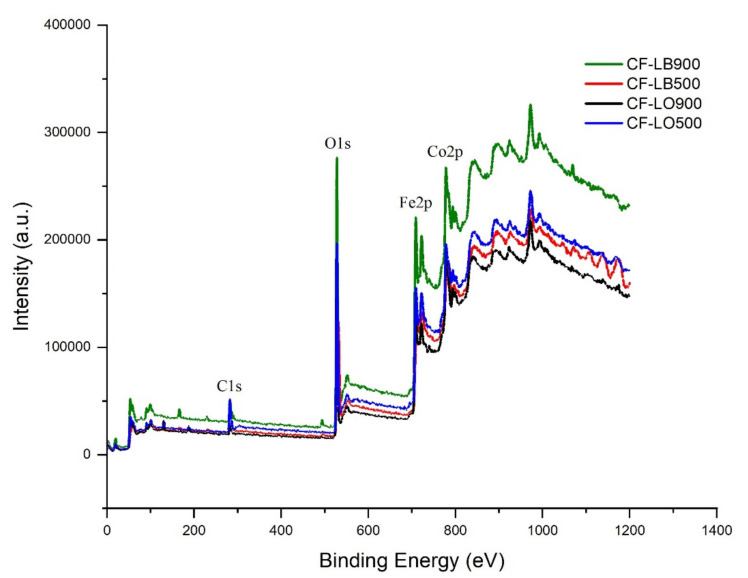
XPS spectra of ferrite-lignin hybrids.

XPS measurements were conducted to provide chemical state information on the materials/electronic state and cations distribution. In the spectra of all materials (Figure 5), the binding energy is located at about 780.1 eV, 710.7 eV, 529.8 eV, and 284.7 eV, which are attributed to the core photoionization signals of Co 2p, Fe 2p, O 1s, and C 1s, respectively, which represents a clear proof of the successful synthesis of ferrite-lignin hybrids. The relative height of the C 1s peak at 284.7 eV confirms that carbon is present in all the materials. 

The O 1s XPS spectra of the hybrids were deconvoluted into four peaks at 530.0 eV, 531.3 eV, 532.06 eV, and 535.13 eV. The peak at 530 eV is assigned to the oxygen ions involved in the crystal lattice of cobalt ferrite (O^spinel^), and that at 531.3 eV corresponds to the O^−^ or O_2_^2−^ species adsorbed on the surface, related to the oxygen vacancy inherent to the surface. The peak at 532.2 eV corresponds to the adsorbed water (the O–H bond) [40]. The peak at 535.13 eV is associated with the carbon single-bonded to the hydroxyl group (C–OH).

On the the Fe 2p spectrum, the peak at a binding energy of 710.2 eV is related to the trivalent iron at the octahedral sites of the spinel ferrite [41], while the 2p_3/2_ peak is deconvoluted into two peaks at binding energies of 711.7 and 713.3 eV, suggesting a multiple characteristic of Fe^3+^ oxidation state in tetrahedral sites.

The Co 2p XPS spectra evidenced four peaks. The peak located at 780.2 eV is attributed to the Co 2p_3/2_ in the octahedral site, while the peak at 782.4 is associated with tetrahedral Co^2+^. The peak at 785.50 eV can be assigned to the interaction of the Co atom and the hydroxyl species in this ferrite. 

**Figure 6:**
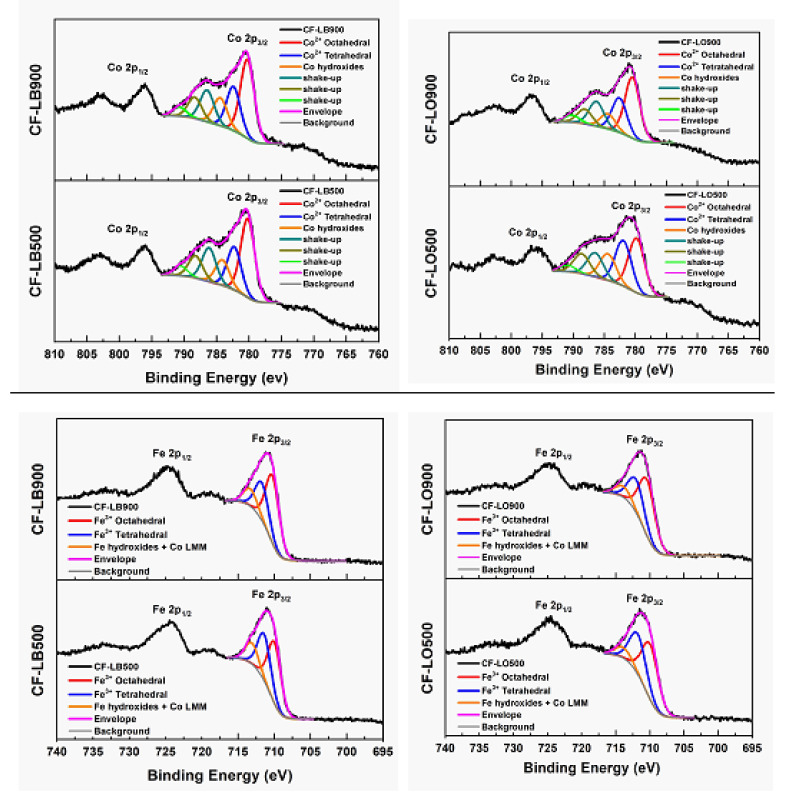
Deconvolution of Co and Fe XPS spectra of the hybrids samples obtained at 500 °C (**bottom**) and at 900 °C (**top**).

According to XPS data (Table 3, Figure 6), a different occupancy of cations at octahedral and tetrahedral sites occurred upon the increase in temperature. The relative contributions to the overall intensity of iron, as well as that of cobalt, in octahedral sites increased, and both have a close relation to each other.

### 3.7. Magnetic Properties

**Figure 7:**
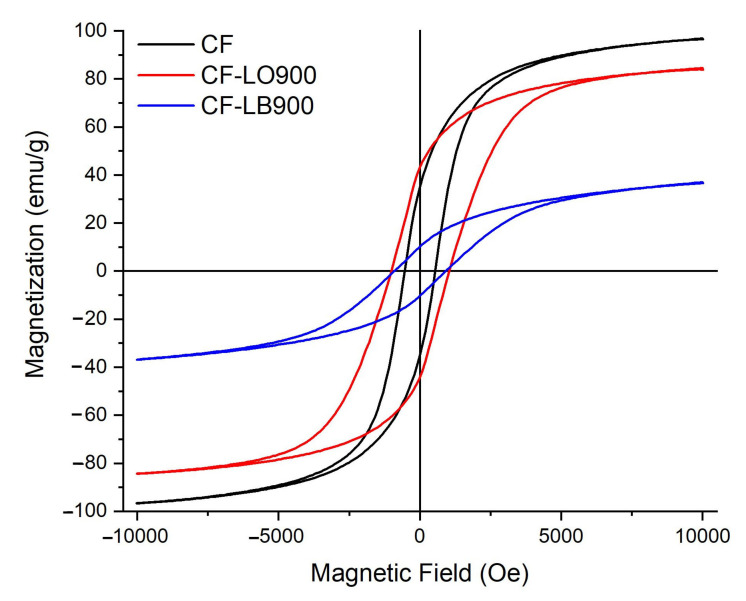
Magnetization as a function of applied field (M−H) hysteresis curves obtained for ferrite and hybrid materials, respectively.

Figure 7 displays the saturation magnetization as a function of the magnetic field (H), measured at room temperature for hybrid materials obtained at 900 °C while the magnetic parameters, namely magnetization measured at *H*_max_ (*M*_s_), coercivity (*H*_c_), and remanent magnetization (*M*_r_), are summarized in Table 4. It is well known that the CoFe_2_O_4_ ferrite is a soft ferromagnetic material and, according to the obtained results, it presented the highest magnetization.

There could be several reasons for the differences between the two hybrid materials, such as the higher crystalline quality of the CF-LO900 particles, the particles and surface sizes, the cationic distribution, and the anisotropy. Moreover, Jeong et al. [42] demonstrated that the magnetic properties are highly volume- and temperature-dependent due to the collective interaction of the magnetic dipoles. Therefore, in our case, it is clear that the calcination temperature of the two types of lignins influenced the surface to volume ratio, with a direct effect on the surface canting. For the CF-LB900, with a larger surface area of 7.58 m^2^ g^−1^, the surface energy and tensions are higher, which lead to lattice distortion, depending on the degree of spinel inversion, and thus a smaller magnetization [43].

According to Samad et al. [44], the magnetic properties of spinel ferrites depend on the distribution of the cations on the tetrahedral and octahedral sites of the spinel structure. In our materials, there are twice as many octahedrally as tetrahedrally coordinated transition metal ions in the structure, which means that the magnetic properties of ferrite-lignin hybrids are related to the choice of the divalent cation, Co^2+^, and the distribution of the cationic species, Co^2+^ and Fe^3+^, between the crystallographic sites in the spinel structure. As the Hc value is the main parameter to identify the possible magnetic applications of these materials, it is worth mentioning that the hybrid comprising LO lignin presented the best magnetic properties. This could be correlated with the specific properties of organic lignin, which has low molecular weight, high chemical purity, and a relatively narrow molecular weight distribution [45] as compared to Lignoboost lignin, and could also influence the Hc value. 

The Hc values of the materials decreased with the grain size increasing above the domain size, according to the D6-power law [46]. However, the Hc value of the CF-LO900 is in the range of 1000 to 1500 Oe, as previously reported [47,48]. Moreover, at the moment it is not clear in which way the coercivity of lignin hybrids is affected by the anisotropy field. According to V. Bartůněk et al., the recorded magnetic behavior makes cobalt ferrite–lignin hybrids promising candidates for medicinal applications [49]. Some authors [50,51] confirmed that ferrite lignin delays the release of ketokonazole from a polysaccharide-polyurethane matrix. Dai et al. reported [52] on the synthesis of lignin nanoparticles and their further use as a green carrier for the sustained release of Resveratrol^®^. In vitro and in vivo studies of the developed system, containing or not containing magnetic particles, evidenced no adverse effects on cells or mice. Other authors [53,54] have studied the effect of lignin nanoparticles in anti-cancer drug delivery, in combination with metal nanocarriers. They reported that the formulated nanocarriers presented low cytotoxicity and an antiproliferative effect on different cancer cell lines. Taking into account the very strict regulations with respect to medically administered substances, more studies are needed before proceeding with clinical trials.

## 4. Conclusions

The paper demonstrates the potential of lignin in the development of cobalt ferrites-lignin hybrids using a simple combustion technique. 

The evaluation of the developed materials by different techniques (FT-IR, XRD, SEM and EDX, XPS, and VSM) has evidenced the formation of cobalt ferrite-lignin hybrids with a spinel structure. It was found that their particle sizes and crystallite size have increased with an increment in the calcination temperature. A different occupancy of cations at octahedral and tetrahedral sites also occurred upon the increase in temperature. SEM images revealed the occurence of various clusters and particles agglomerations with the temperature increase, which is consistent with the XRD results. The hybrids comprising LO lignin presented the best magnetic properties. 

The lignin needed for the designed hybrids is a by-product of the paper and pulp industry, which means that ferrite-lignin hybrids could be produced at a low cost, as compared to other synthesis routes. However, research in this field is still in the early stages, and many aspects need to be investigated for the successful exploitation of lignin in high-value applications.

## Figures and Tables

**Table 1:** Statistical numeric indicators of the wppf (Rwp, S and χ²) and the calculated lattice parameter (a) and unit cell volume (*V*).

Sample	Rwp (%)	S	χ²	a (Å)	V (Å^3^)	Crystallite Size, (Å)
CF-LB500	4.96	2.044	4.178	8.4104 ± 0.0018	594.914	119
CF-LB900	2.66	1.0920	1.1924	8.38490 ± 0.00017	589.514	615
CF-LO500	1.92	0.8064	0.6503	8.3816 ± 0.00010	588.827	76
CF-LO900	3.22	1.1147	1.2425	8.3858 ± 0.0003	589.708	332

**Table 2:** DVS parameters of lignin hybrids.

Sample	Water Sorption Capacity, (% d.b.)	Average Pore Size, (nm)	BET	
			Area, (m^2^ g^−1^)	Monolayer, (g g^−1^)
CF-LB500	7.50	1.04	144	0.03410
CF-LB900	2.07	5.48	7.58	0.00210
CF-LO500	5.00	3.29	30.5	0.00879
CF-LO900	0.84	4.65	3.62	0.00103

**Table 3:** Detailed XPS data for ferrite-lignin hybrids.

Sample	CF-LB500			CF-LB900			CF-LO500			CF-LO900		
Element	BE (eV)	Assignment	Conc. (%)	BE (eV)	Assignment	Conc. (%)	BE (eV)	Assignment	Conc. (%)	BE (eV)	Assignment	Conc. (%)
C 1s	285 *	C-C/C-H	69.07	285 *	C-C/C-H	61.88	285 *	C-C/C-H	55.99	285 *	C-C/C-H	61.39
	286.5	C-O	13.46	286.5	C-O	12.82	287	C-O	17.26	286.5	C-O	11.60
	288.5	C=O/O-C=O	17.47	288.8	C=O/O-C=O	25.30	288.9	C=O/O-C=O	26.75	288.8	C=O/O-C=O	27.01
O 1s	529.9	O oxides	58.05	530	O oxides	63.74	529.6	O oxides	54.21	530	O oxides	48.12
	531.2	O hydroxides	21.60	531.3	O hydroxides	18.70	531.1	O hydroxides	36.08	531.3	O hydroxides	36.28
	532.1	C=O/O-C=O	16.01	532.1	C=O/O-C=O	13.73	532.5	C=O/O-C=O	8.27	532.4	C=O/O-C=O	12.29
	533.3	C-O	4.34	533.3	C-O	3.83	533.7	C-O	1.44	533.8	C-O	3.31
Fe 2p	710	Fe^3+^ Octahedral	47.47	710.2	Fe^3+^ Octahedral	55.09	710	Fe^3+^ Octahedral	52.07	710.4	Fe^3+^ Octahedral	56.11
	711.4	Fe^3+^ Tetrahedral	36.30	711.7	Fe^3+^ Tetrahedral	30.80	711.8	Fe^3+^ Tetrahedral	38.10	712	Fe^3+^ Tetrahedral	33.11
	713.1	Fe hydroxides and Co LMM	16.23	713.3	Fe hydroxides and Co LMM	14.11	713.9	Fe hydroxides and Co LMM	9.83	713.9	Fe hydroxides and Co LMM	10.78
Co 2p	780.2	Co^2+^ Octahedral	36.97	780.3	Co^2+^ Octahedral	37.12	779.8	Co^2+^ Octahedral	30.73	780.5	Co^2+^ Octahedral	38.79
	782.3	Co^2+^ Tetrahedral	20.23	782.4	Co^2+^ Tetrahedral	20.72	782	Co^2+^ Tetrahedral	25.71	782.6	Co^2+^ Tetrahedral	21.89
	784.1	Co^2+^ hydroxides	12.01	784.4	Co^2+^ hydroxides	13.09	784.4	Co^2+^ hydroxides	15.00	784.4	Co^2+^ hydroxides	9.69
	786.2	shake-up	15.77	786.5	shake-up	14.82	786.5	shake-up	13.54	786.3	shake-up	15.76
	788.3	shake-up	10.45	788.4	shake-up	9.95	788.6	shake-up	11.38	788.2	shake-up	9.28
	790.5	shake-up	4.57	790.7	shake-up	4.30	790.9	shake-up	3.63	790.2	shake-up	4.59

* reference peak; BE = binding energy; Conc. = relative concentration.

**Table 4:** Magnetic parameters of ferrite-lignin hybrids synthetized at 900 °C.

Sample	Coercivity, *H*_c_ (Oe)	Remanent Magnetization, *M*_r_ (emu/g)	Magnetization Measured at *H*_max_, *M*_s_ (emu/g)
CoFe_2_O_4_ (CF)	543.80	34.9	82.69
CF-LB900	854.60	10.10	26.81
CF-LO900	1048.8	43.39	72.88

## Data Availability

Data sharing not applicable.
